# Senescence-specific translation dysregulation desensitizes cells to stress by inhibiting activation of the integrated stress response

**DOI:** 10.1101/2023.04.12.536613

**Published:** 2023-04-12

**Authors:** Matthew J. Payea, Showkat A. Dar, Carlos Anerillas, Jennifer L. Martindale, Cedric Belair, Rachel Munk, Sulochan Malla, Jinshui Fan, Yulan Piao, Xiaoling Yang, Abid Rehman, Nirad Banskota, Kotb Abdelmohsen, Myriam Gorospe, Manolis Maragkakis

**Affiliations:** Laboratory of Genetics and Genomics, National Institute on Aging, Intramural Research Program, National Institutes of Health, Baltimore, MD 21224, USA

## Abstract

Senescence is a state of indefinite cell cycle arrest associated with aging, cancer, and age-related diseases. Here, using label-based mass spectrometry, ribosome profiling and nanopore direct RNA sequencing, we explore the coordinated interaction of translational and transcriptional programs of human cellular senescence. We find that translational deregulation and a corresponding maladaptive integrated stress response (ISR) is a hallmark of senescence that desensitizes senescent cells to stress. We show that senescent cells maintain high levels of eIF2α phosphorylation, typical of ISR activation, but translationally repress the stress response transcription factor 4 (ATF4) by ineffective bypass of the inhibitory upstream open reading frames. Surprisingly, ATF4 translation remains inhibited even after acute proteotoxic and amino acid starvation stressors, resulting in a highly diminished stress response. Furthermore, absent a response, stress exacerbates the senescence secretory phenotype and inflammatory pathways thus acting as a possible mechanistic link to disease. Our results reveal a novel mechanism that senescent cells exploit to evade an adaptive stress response and remain viable.

## Introduction

Cellular senescence is a state of virtually permanent cell cycle arrest that typically occurs in response to sublethal genomic damage. Senescence is expected to primarily guard against carcinogenesis by forcing cells with potentially tumorigenic mutations out of the cell cycle^1,2^, though other functions for senescence in development^3^ and wound healing^4,5^ have also been described. However, senescent cells are also detrimental to human health as their prevalence increases during aging^6^ and their accumulation is linked to several diseases ^7–9^. The detrimental trait of senescent cells on organismal health is primarily attributed to the senescence associated secretory phenotype (SASP), which describes the dramatic increase in secretion of interleukins, cytokines, and other proteins^10–14^. The link between senescence and age-related diseases has been further established by advancements in senolytics, a class of senescent-specific cytotoxic drugs, that have been used to show that selective clearance of senescent cells is associated with improvements in health^15–17^. Thus, there is a growing appreciation for a seno-centric framework of aging^18^, in which senolytic therapies can serve a unified approach for aging intervention^19–23^.

Since senolytics would primarily serve as quality-of-life therapies, it is important to identify senescence-specific changes to cellular function that can be exploited for maximum selectivity. The current knowledge on the biology of senescence benefits from high-throughput analyses that have established several key components of the senescent transcriptome and secretome^11,12,24,25^, but comparatively less is known about the senescent proteome^26–28^ and the involved translation regulatory mechanisms^29^.

Here we coupled Tandem-Mass Tag (TMT) mass spectrometry, ribosome profiling, and full-length, direct RNA nanopore sequencing (dRNA-Seq) in senescent, quiescent, and cycling states to discover senescence-specific regulation of translation and the proteome that directly dictates cell survival and secretory programs in human cellular senescence. Our analysis identified that senescent cells possess unique increases in ER stress proteins, decreased spliceosome proteins, and a global reduction in translating ribosomes. Interestingly, they also display a stable increase in eIF2α phosphorylation, a critical integrated stress response (ISR) marker, surprisingly without downstream induction of the key ISR transcription factor ATF4.

We determined that this resistance to ATF4 expression in senescence is a consequence of the decreased competition for ternary complex due to a widespread decrease in translation that leads to inefficient ribosome bypass of upstream Open Reading Frames (uORF) in ATF4. Consequently, we find that the reduced efficacy of ATF4 expression causes senescent cells to be globally resistant to ISR activation under both proteotoxic stress and amino acid starvation. Surprisingly, senescent cells maintain increased eIF2α-P even 20h after stress has been removed, accompanied by a durable transcriptional increase in several inflammatory proteins. Our work provides the most complete profiling of the intersection between transcriptome, translatome, and proteome for senescence, and identifies a fundamental cellular mechanism that confers resistance to stress response.

## Results

### Cellular senescence is characterized by persistent ER stress signaling but post-transcriptional inhibition of ATF4 activation

**Figure 1.**
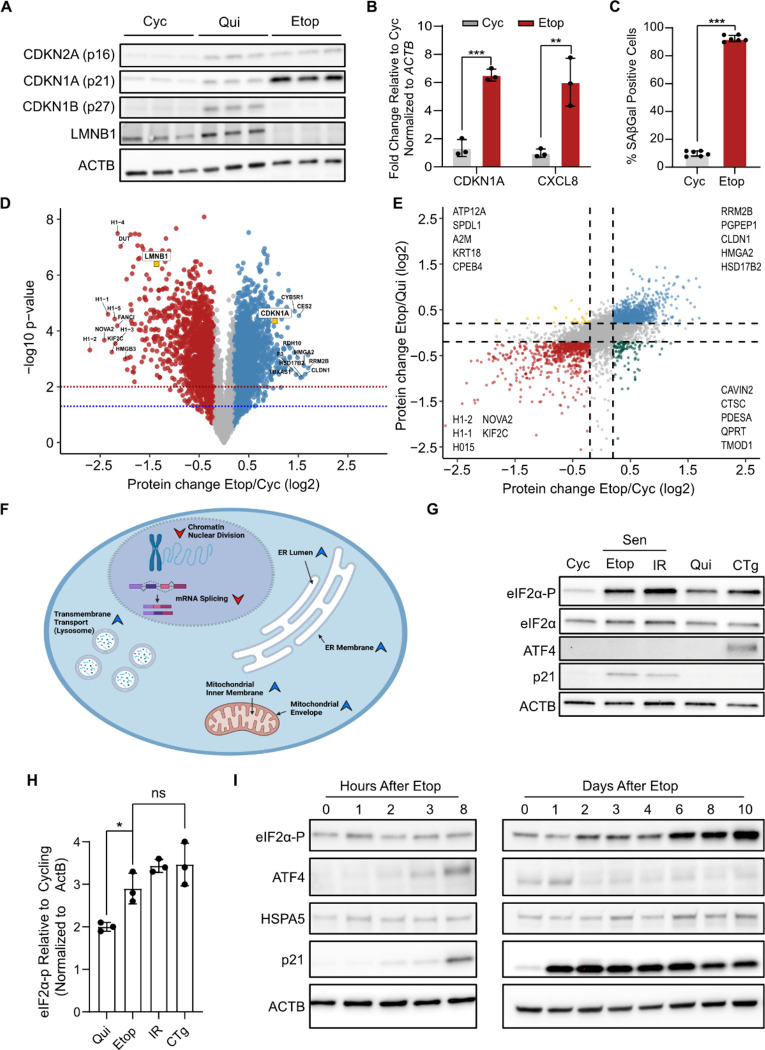
Cellular senescence is characterized by persistent ER stress signaling but no downstream ATF4 activation **(A)** Western blot analysis of cell cycle arrest markers p21 (CDKN1A), p16 (CDKN2A) and p27 (CDKN1B) and nuclear protein Lamin B1 (LMNB1) for etoposide induced senescent, quiescent, and cycling cells. ACTB is used as control. **(B)** RT-qPCR analysis of *CDKN1A* and *CXCL8* mRNAs from cells analyzed in (A). Significance determed by t-test with n=3 (**= pvalue<0.01; *** = pvalue < 0.001) **(C)** Quantification of beta galactosidase staining (SA-βGal) of etoposide treated and cycling cells (*** = pvalue < 0.001). **(D)** Volcano plot of Etop/Cyc differential protein expression analysis. Statistically significant (p-value ≤ 0.05 by two-tailed t-test) log2FC values are colored as follows: log2FC ≥ 0.2, blue; log2FC ≤ −0.2, red; log2FC between −0.2 and 0.2, grey. **(E)** Scatterplot of protein fold-change values from the Etop/Cyc (x-axis) and Etop/Qui (y-axis) comparisons. Dotted lines mark 0.2 and −0.2 log2FC in both comparisons. Quadrants are annotated with the top 5 most changed proteins, with respect to Etop/Cyc. **(F)** Summary schematic of GSEA for senescence-specific increased and decreased proteins. Red arrows indicate annotation is suppressed in senescence and blue arrows indicate annotation is increased in senescence. **(G)** Western blot analysis of ISR markers eIF2α phosphorylation (eIF2α-P) and ATF4; cells analyzed are cycling (Cyc), etoposide-induced senescent (Etop), ionizing-radiation induced senescent (IR), contact-inhibited quiescent (Qui), and thapsigargin-treated (CTg, 25 nM 3h). **(H)** Quantification of eIF2α-P signal relative to Cyc cells for Etop, IR, Qui, and CTg cells determined from western blot analysis in triplicate; normalized against ACTB protein expression. **(I)** Western blot of lysates harvested at indicated time points after initial etoposide treatment (left, hours; right, days).

To perform a direct and quantitative comparison of the senescent, cycling, and quiescent proteomes, we used TMT isobaric labeling and mass spectrometry using IMR-90 human lung fibroblasts. We chose a DNA-damage induced model of senescence for study, which was generated through treatment of cells with the topoisomerase II inhibitor etoposide as previously described ^30,31^. Senescent cells were compared to both young dividing cells (Cyc) and contact-inhibited quiescent cells (Qui), to control for changes associated with general cell cycle arrest. We validated the senescence phenotype of Etop cells using a multi-marker workflow^32^. As expected, western blot analysis indicated Etop cells had increased expression of cell-cycle inhibitors (p16^INK4A^, p21^CIP1^) and decreased expression of LMNB1 (Fig. 1A). Similarly, RT-qPCR analysis showed increased expression of *CDKN1A* (p21^CIP1^) and the SASP factor *CXCL8* (Fig. 1B). Senescent cells also showed high positivity in a senescence-associated β-galactosidase activity (SA-βGal) assay compared to cycling cells, further confirming the senescence phenotype (Fig. 1C, Fig. S1A).

Whole-cell protein lysates from the Etop, Cyc, and Qui cells were harvested in biological triplicate, processed for TMT-multiplex labeling, fractionated, and then analyzed by LC/MS/MS. We identified 6,254 proteins across all 9 libraries after filtering (Table S1, see methods). Principal component analysis (PCA) showed that the proteomes of Cyc, Qui, and Etop cells were highly distinct whereas replicates were closely associated (Fig. S1B). We performed differential protein expression analysis for Etop/Cyc and Etop/Qui comparisons and defined a threshold for significant biological change that was retrospectively determined to be at least ~2-fold by western blot analysis (Fig. S1C, see methods). Our analysis identified several proteins with substantial change in both comparisons, including expected changes in the senescence markers relative to both Cyc and Qui cells (Fig.1D, Fig. S1D).

To identify robust protein expression changes that were unique to the senescence state, we selected for proteins that were significantly changed in Etop compared to both Cyc and Qui cells. This revealed 866 proteins that were uniquely decreased in senescence and 1,112 that were uniquely increased (Fig. 1E, Table S1). Gene Set Enrichment Analysis (GSEA)^33^ identified several trends associated with previously established senescence phenotypes^1,32^, as well as two novel and significant areas of senescence-specific change: suppression of mRNA splicing and activation of endoplasmic reticulum (ER) proteins (Fig. 1F, Fig. S1E). Further enrichment analysis with the Kyoto Encyclopedia of Genes and Genomes (KEGG)^34^ confirmed the spliceosome and ER processing pathways as areas of strong regulation (Fig. S1F,G), suggesting that changes in both of these processes may be hallmarks of senescence.

Notably, we observed increases in PERK (eIF2AK3) (Fig. S1F), an ER stress sensing kinase that phosphorylates the translation initiation factor eIF2α (eIF2α-P) to both reduce protein synthesis and promote expression of the multi-functional transcription factor ATF4 ^20^. We therefore measured eIF2α-P and ATF4 levels to test whether the ISR and unfolded protein response (UPR), which co-activate to resolve proteotoxic stress, were basally activated in senescent cells. We used Etop cells and an additional DNA-damage induced senescent model: ionizing radiation induced (IR) senescence. As a reference for ER stress, we compared Etop and IR cells to cells treated with the ER stress inducer thapsigargin (Tg)^35^, referred to as CTg cells hereafter. Etop and IR cells had distinctive increases in eIF2α-P, which were highly reproducible and similar in magnitude to CTg cells. However, in contrast to CTg, we found that surprisingly neither Etop nor IR cells expressed ATF4 (Fig. 1G,1H). RT-qPCR analysis showed no reduction in *ATF4* mRNA in senescence compared to Cyc cells contrary to what has been previously described for UV treated cells ^36,37^ (Fig. S1H), which indicated ATF4 was likely suppressed by an unknown post-transcriptional mechanism.

Since the senescence phenotype has been observed to have high heterogeneity with respect to both method of induction and cell type ^11,24,25^ we examined whether increased eIF2α-P without ATF4 expression was generalizable to other models. We found that Etop treatment of WI-38 lung fibroblasts, BJ foreskin fibroblasts, and HUVEC vein endothelial cells produced similar increases in eIF2α-P without ATF4 expression (Fig. S1I). Similarly, we saw that replicative exhaustion of IMR-90 cells also produced the same phenotype (Fig. S1J). The induction of senescence occurs in phases ^31,38^ and so we tested whether ATF4 was expressed earlier during the establishment of senescence. Interestingly, we found that while eIF2α-P levels steadily increased over time, ATF4 expression only briefly increased at induction but reduced shortly after (Fig. 1I). Thus, our results show that senescence is generally associated with a continual source of ER stress that chronically induces eIF2α-P but with a progressive insensitivity to it that limits induction of ATF4 post-transcriptionally.

### Ribosome uORF bypass is not activated in senescent cells

Phosphorylation of eIF2α in the ISR serves primarily to limit availability of the ternary complex (eIF2α-GTP-tRNA_i_^Met^), which results in decreased translation initiation and increased 40S ribosomal subunit scanning ^20^. Therefore, while increased eIF2α-P levels decrease translation globally, they also promote ribosome bypass of an inhibitory upstream open reading frame (uORF) in favor of the main ATF4 ORF. The disconnect between eIF2α-P levels and ATF4 protein expression suggested that eIF2α-P was not sufficiently limiting the ternary complex availability for uORF bypass in senescence.

**Figure 2.**
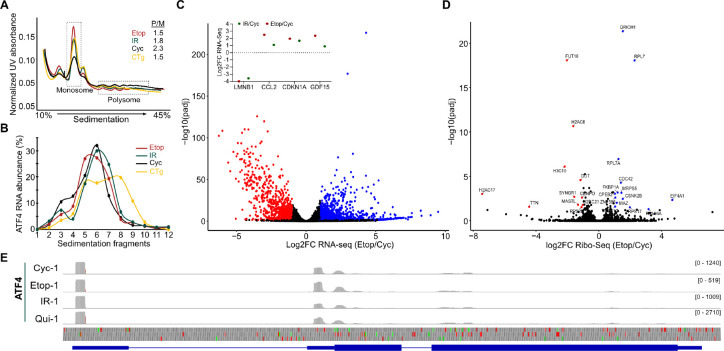
Ribosome sequencing shows that uORF bypass is not activated in senescent cells. **(A)** Polysome profiling traces on a 10–45% sucrose gradient for lysates from indicated cell types after treatment with 0.1 mg/mL. The ratio of polysome/monosome fractions (P/M) are quantified and shown in inset. **(B)** RT-qPCR analysis of ATF4 levels for RNA extracted from the polysome profiling experiment described above; increasing fragments correspond to higher density sucrose and polysomes. **(C)** Volcano plot of Etop/Cyc differential RNA-seq expression analysis. Statistically significant (adjusted p-value ≤ 0.05 by two-tailed t-test) log2FC values are colored as follows: log2FC ≤ −1, red; log2FC ≥ 1, blue; log2FC between −1 and 1, black. Inset indicates log2FC values for senescence markers in the Etop/Cyc (shown) and IR/Cyc comparisons. **(D)** Volcano plot of Etop/Cyc differential translation efficiency (see methods) from ribosome sequencing. Statistically significant (adjusted p-value ≤ 0.05 by two-tailed t-test) log2FC values are colored as follows: log2FC ≤ −1, red; log2FC ≥ 1, blue; log2FC between −1 and 1, black. **(E)** IGV mapping of Ribo-seq reads for the ATF4 gene locus of the hg38 human genome release. Increased density indicates ribosome occupancy.

To test this hypothesis, we performed polysome profiling to probe the translational status of ATF4 mRNA. We performed polysome profiling on Cyc, Etop, IR, and CTg cells, and found that both Etop and IR cells had profiles that strongly resembled that of CTg cells consistent with ISR activation, with a characteristic increase in non-translating monosomes and decrease in translating polysomes (Fig. 2A). RT-qPCR analysis of our polysome profiling fractions showed that *ATF4* mRNA was predominantly in the non-translating monosome fraction of senescent and Cyc cells, while CTg cells showed a clear shift toward the polysomes (Fig. 2B). This result shows that the ATF4 mRNA is not engaged with ribosomes as would have been expected under high eIF2α-P levels that would permit uORF bypass, We further tested the contribution of eIF2α-P in senescence by treating cells with the ISR inhibitor (ISRIB) drug, which suppresses the effects of eIF2α-P on translation. Treatment of CTg cells with ISRIB produced a substantial reduction in monosome accumulation but showed very little effect on Etop cells (Fig. S2A), thus showing that eIF2α-P has little effect on translation in senescence.

To further test the translation dynamics on ATF4 and to globally assess why eIF2α-P may not be functioning as expected in senescence, we performed ribosome sequencing (Ribo-Seq) of Etop, IR, Qui, and Cyc cells and coupled that with RNA sequencing (RNA-seq) measured in parallel from a single lysate. We calculated translation efficiency by quantifying ribosome footprints relative to mRNA levels as previously described ^40^. We found good correlation between replicates of each condition in both the Ribo-Seq and RNA-seq libraries (Table S2, Fig. S2B). PCA showed that Etop and IR datasets were closely associated while also being distinct from the Cyc and Qui conditions (Fig. S2C). Differential RNA-seq analysis captured several markers of senescence as previously described ^24,25^, and confirmed our finding that *ATF4* mRNA levels are not substantially changed in senescence relative to Cyc cells (Fig. 2C, Table S2). Similarly, GSEA identified gene expression patterns frequently ascribed to the senescence phenotype (Fig. S2D). We next identified transcripts with detectable changes to translational efficiency relative to Cyc cells. We found that both Etop and IR cells had very few genes (25 and 29 genes, respectively) with a statistically significant (adjusted p-value ≤ 0.05) change in translation efficiency, though both were greater than what was observed in Qui cells (5 genes) (Fig. 2C, Fig. S2E). Consistent with our western and polysome analysis, we found no significant difference in the translation efficiency of *ATF4* in either of the four conditions (Table S2). Further examination of ribosome footprint distribution on *ATF4* mRNA showed that ribosomes were predominantly located in the uORFs rather than in the coding region for Etop and IR cells (Fig. 2D). These results confirmed that senescent cells were not permitting uORF bypass for ATF4 despite elevated eIF2α-P.

### Global decrease in translating ribosomes inhibits the ISR in senescence

**Figure 3.**
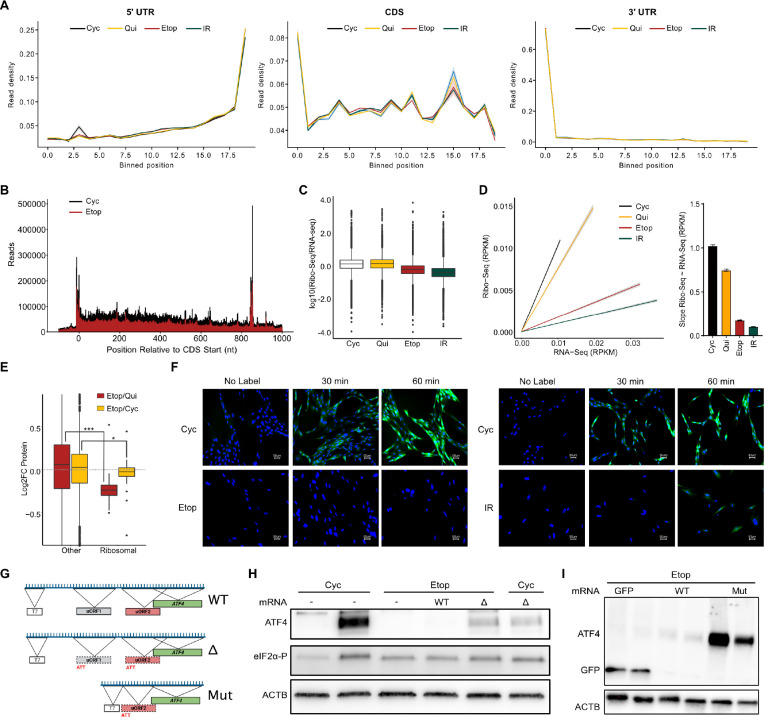
A global decrease in translating ribosomes inhibits the ISR in senescence. **(A-B)** Metaplot analysis of Ribo-seq reads mapped to the transcriptome. In **(A),** the 5’ end coordinates of aligned reads are transformed to binned coordinates that effectively represent percentage of total gene length, and in **(B)**, reads are aligned relative to annotated CDS start sites. **(C)** Boxplot of log_10_(Ribo-seq/RNA-seq) reads as determined by RPKM analysis (see methods); boxplot inset of the same data with median of each sample marked. **(D)**. Linear regression analysis of Ribo-seq~RNA-seq reads (left) and barplot comparing slopes obtained with standard error (right). **(E)** Boxplot of ribosomal protein (RProtein) abundances from mass spectrometry data for Etop/Cyc (red) and Etop/Qui (yellow) comparisons. The RProtein group is significantly reduced compared to the total group of proteins in each comparison by student’s t-test (*= pvalue<0.01; *** = pvalue < 0.001). **(F)** Representative immunofluorescence images (n=4) from Etop and Cyc cells pulsed with 50 μM methionine analogue and fixed after indicated time. **(G)** Diagram of T7-ATF4 mRNA constructs for transfection; dotted lines indicate uORF initiation site has been mutated as shown. **(H-I)** Western blot analysis of transfected T7 transcribed *ATF4* mRNA.

The lack of uORF bypass we observed in senescence indicated that eIF2α-P levels were not effectively increasing 40S scanning. We considered senescent cells may have a separate global translational change that suppressed the effects of eIF2α-P, and which would not be easily detectable from measurements of translation efficiency. To test this we performed metagene analysis to identify potential changes in the distribution of ribosome footprints along mRNAs. We found that senescent cells possessed a minor bias toward the 5’ end of the coding sequence (CDS) compared to Cyc and Qui cells (middle panel) and were nearly identical in the 5’ UTR and 3’ UTR regions (Fig. 3A). Similarly the overall ribosome footprint distribution within 1000 nts of the start site did not indicate any substantial change between our libraries. However, senescent cells had far less reads in total over the same window (Fig. 3B, Fig. S3A), suggesting fewer translating ribosomes in total during senescence. Indeed, after normalizing for RPKM, we found that the ratio of Ribo-seq/RNA-seq reads for both Etop and IR cells was substantially less compared to both Cyc and Qui cells (Fig. 3C, Table S2, see methods). Linear regression analysis further showed that senescent cells had ~4 times less ribosomes per unit of RNA compared to either Cyc or Qui cells (Fig. 3D).

Our ribosome sequencing data thus strongly suggested that there were fewer translating ribosomes in senescent cells, indicating dramatic decreases in senescent translation machinery. A search of translation factors within our mass spectrometry dataset identified several as uniquely decreased in senescence (Table S2). Most impressively, ribosomal proteins experienced a statistically significant (p-value ≤ 0.05) group-wide reduction in both the Etop/Cyc and Etop/Qui comparisons (Fig. 3E). Decreased ribosomal proteins were consistent with the reduction in ribosome footprints we observed, as well as broad decreases in rRNA biogenesis in senescence ^41,42^. Our results predict that senescent cells would have markedly reduced total protein synthesis which we verified by measuring the rate of nascent protein production after pulsing with a methionine analogue. We found that both Etop and IR cells produced far less protein (green signal) over a 60-minute interval compared to Cyc cells (Fig. 3F, S3B), consistent with a senescence-associated global decrease in translating ribosomes.

A reduction in translating ribosomes presented a mechanistic explanation for why the uORF bypass of ATF4 would be ineffective in senescence. ATF4 expression depends on the dynamic balance of ternary complex availability and ribosome demand to initiate translation. Under high ribosome load and reduced ternary complex availability, 40S scanning through the inhibitory uORF is promoted. However, under a senescence-associated ribosome decrease, the ternary complex availability would effectively appear increased, thus suppressing 40S scanning. In fact, previous work has suggested that the eIF2α pool is typically used at capacity in cells^43^, and while our data show decrease in ribosome footprints and ribosomal proteins, we observed no change in eIF2α protein expression (Table S1), indicating it is at excess of translation during senescence. A core prediction of our model is that senescent cells should be capable of expressing ATF4 if the inhibitory uORF is removed. We tested this by generating constructs with ATF4 downstream of a T7 promoter using intact (WT), missense mutation carrying (ΔuORF), and 5’ UTR shortened past uORFs (Mut) (Fig. 3G). As predicted by our model, we found that transfection of WT *ATF4* produced very little protein expression, while ΔuORF produced substantially more (Fig. 3H). Transfection of the Short-*ATF4* construct resulted in even further increase (Fig. 3I), possibly due to elimination of uORF0 that we had observed a substantial amount of ribosome footprint density on (Fig. 2D). Collectively, our data show that senescent cells have fewer translating ribosomes and that this causes decreased uORF bypass with direct consequences for ATF4 translation inhibition and the ISR.

### Senescent cells fail to activate the ISR in response to acute stress

**Figure 4.**
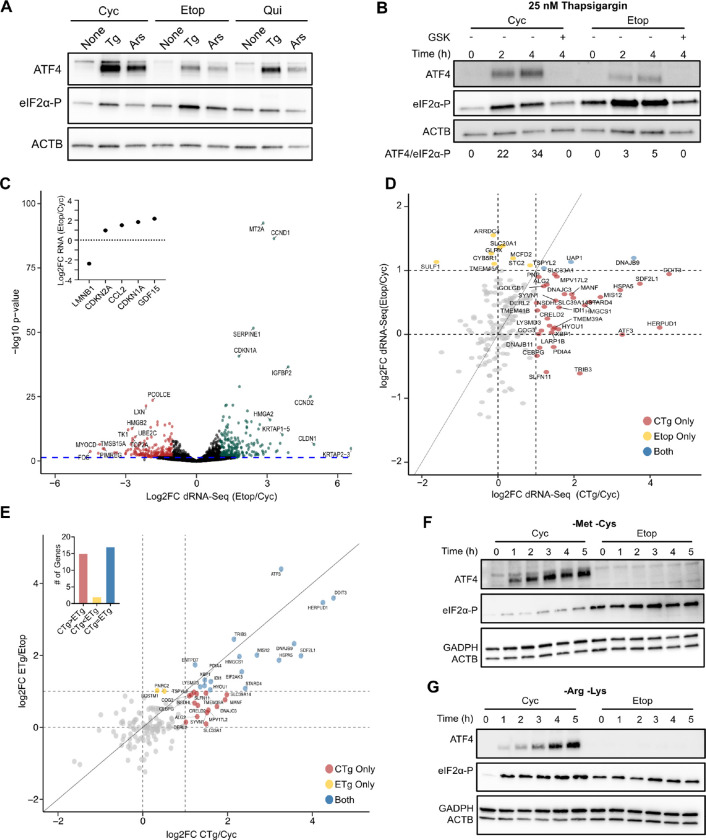
Senescent cells fail to activate the ISR in response to acute stress. **(A)** Western blot of Etop, Cyc, and Qui cells treated for 3 h with vehicle (none), 25nM Tg (Tg), or 250 μM sodium arsenite (Ars). **(B)** Western blot of Etop and Cyc cells treated with 25 nM Tg and harvested after indicated times; eIF2AK3 inhibitor (GSK, 550 nM) added concurrently with Tg for indicated lane. The ATF4/eIF2α-P ratio is determined by intensity-based quantification and rounded to nearest whole number. **(B)** Western blot of Cyc and Etop cells treated with 25nM thapsigargin for 3h in biological triplicate as indicated. **(C)** Volcano plot of nanopore-based direct RNA sequencing (dRNA-seq) abundance changes for Etop vs. Cyc cells. RNAs with statistically significant (adjusted p-value ≤ 0.05 by two-tailed t-test) abundance increase in red, decrease in green. The top ten genes, increased or decreased, are annotated. **(D-E)** Scatter plot of differential gene expression for previously described ER-stress activated genes^21,47,48^. Dots are colored based on whether they are at least 2-fold increased (above dashed lines) in the indicated comparisons (exclusive or both). For **(E)**, sum of total genes in each category shown as inset. **(F-G)** Western blot of Cyc and Etop cells starved for indicated times in culture media lacking amino acids, for **(F)** Methionine and Cysteine (-Met -Cys) and for **(G)** Arginine and Lysine (-Arg -Lys).

Our model also suggested that senescent cells would be less responsive to additional stressors registered by the ISR. We thus compared the ability of senescent, cycling, and quiescent cells to express ATF4 after acute proteotoxic and oxidative stress through treatment with Tg and sodium arsenite (Ars), respectively. We found that while treatment of Cyc and Qui cells with either stressor induced a strong increase, Etop cells showed substantial resistance to ATF4 expression despite higher than Cyc and Qui cells eIF2α-P levels (Fig. 4A). We next tested whether the kinetics of ISR activation had changed in senescence. Treatment of Cyc cells with Tg produced a rapid increase in eIF2α-P and concurrent ATF4 protein expression, which was suppressed by addition of the eIF2AK3 inhibitor GSK2656157^44^ (Fig. 4B, Fig. S4A). Conversely, Tg treatment of both Etop and IR cells again elicited a far weaker expression of ATF4, despite substantial increases in eIF2α-P (Fig. 4B, Fig. S4A). Quantification of the ATF4/eIF2α-P signal ratio showed that Etop cells were ~7 times less effective at inducing ATF4 expression compared to Cyc cells, despite similar levels of *ATF4* mRNA (Fig. S4B), consistent with our model of an increased eIF2α-P threshold for the ISR in senescent cells.

Our initial screen with Tg and Ars suggested that general cell cycle arrest was not sufficient to cause ISR restriction as Qui cells were able to produce ATF4 in response to Tg and Ars. We additionally compared Tg treatment of Etop, Cyc, and serum-starved quiescent cells and again found that senescent cells were uniquely resistant to expression of ATF4 (Fig. S4C). Previous studies have shown that cells induced to senescence through replicative exhaustion are resistant to the UPR^23^ and so we tested whether the UPR would be similarly inactive for Etop cells treated with Tg. In fact, we observed a complete absence of mature Xbp1 and ATF6 UPR transcription factors ^45^ in Tg treated Etop cells (Fig. S4D), indicating a general senescence-specific restriction in the stress response.

Our results indicated that while senescent cells possess elevated eIF2α-P, they do not activate the downstream transcriptional program of the ISR or UPR. To comprehensively test this, we performed full-length, long-read direct RNA sequencing (dRNA-seq)^46^ of Cyc, Etop, ETg, and CTg cells (Table S3). Our data were highly reproducible across replicates (Fig. S4E) and allowed the simultaneous quantification of the abundance and splicing status of the senescence transcriptome at single-molecule resolution. To our knowledge, there have been no previous analyses of senescent cells using nanopore based sequencing technology and so we first performed differential expression analysis between untreated Etop and Cyc cells. Overall, we quantified 8,587 expressed genes (Table S3, see methods), 271 of which showed statistically significant (adjusted p-value ≤ 0.05) mRNA abundance changes of at least 2-fold, including several senescent markers ^24,25,32^(Fig. 4C, Fig. S4F). Long-read sequencing also allowed us to interrogate features of the mRNAs that are not typically captured in short read RNA-Seq such differences in 5’ end degradation and poly-A tail deadenylation that may indicate changes in mRNA decay^46^. Our data showed little significant change (q-value ≤ 0.05) between Cyc and Etop cells in both features, though we did note a minor trend toward shortened 5’ ends in senescence at a lower threshold of significance (Fig. S4G).

We next interrogated the expression of previously identified targets of the ISR and UPR^21,47,48^ in the Etop/Cyc and CTg/Cyc comparisons. As predicted, we found that while CTg cells displayed 2-fold increases in several of these genes, only few of them were elevated in Etop cells (Fig. 4D). We further examined whether senescent cells would undergo transcriptional changes after Tg treatment. We found that while ETg cells did increase expression over ETop for some of these ISR/UPR genes, the response was highly diminished compared to CTg cells (Fig. 4E), strongly suggesting that the inefficiency of ATF4 expression is indicative of a decreased transcriptional response.

We also tested whether senescent cells would be able to trigger the ISR in response to a different stressor: starvation of amino acids. We grew Cyc and Etop cells in media lacking the amino acids methionine and cysteine (-Met -Cys) and supplemented with dialyzed FBS, which lacks amino acids, to maintain growth rate and accelerate amino acid starvation. We found that senescent cells produced no detectable ATF4 protein even after five hours of amino acid starvation, in stark contrast to Cyc cells that responded within 1h (Fig. 4F). This resistance to starvation was independent of amino acid composition, as starvation of lysine and arginine (-Lys -Arg) in Etop and Cyc cells produced similar results (Fig. 4G). Furthermore, we found that resistance to ATF4 expression extended to IR cells (Fig. S4H) but was not present in Qui cells (Fig. S4I), arguing that the lack of starvation response was a function of senescence and not general to cell cycle arrest.

We again used dRNA-seq to analyze mRNA changes between Etop and Cyc libraries to cells starved of methionine and cysteine (ESv, CSv) (Table S3). As with the response to ER stress, we evaluated the starvation response in ESv and CSv cells by looking for mRNA increases in genes previously identified as upregulated by starvation^49^. We saw that CSv cells produced increases in the greatest number of starvation targets (Fig S4J). Combined, our data strongly suggest that senescent cells are generally resistant to activation of stress response pathways at both the proteomic and transcriptomic level even when challenged by additional stress.

### Unresolved acute stress enhances the senescence associated secretory phenotype

**Figure 5.**
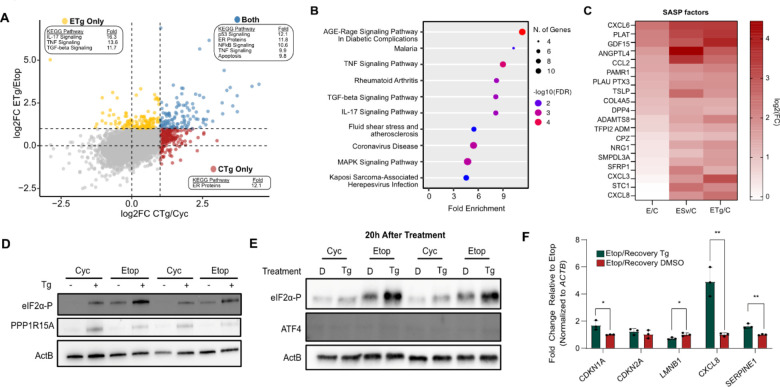
Unresolved acute stress enhances the senescence associated secretory phenotype. **(A)** Comparison of log2FC values for the ETg/Etop and CTg/Cyc comparisons for all detected RNAs; insets show enriched non-disease KEGG pathways for genes in respective quadrant. **(B)** KEGG pathway enrichment analysis of genes observed to be uniquely enhanced by stress. **(C)** Heatmap of genes with annotated secretory function in the stress enhanced gene set; color indicates increasing log2FC for each library relative to cycling cells. **(D)** Western blot analysis of cells treated with or without thapsigargin (Tg; 25 nM, 3h). **(E)** Western blot of cells treated with DMSO (D) or Tg (as above) and then allowed to recover for 20h before harvest. **(F)** RT-qPCR analysis of cells treated in **(E)**, significance assessed by student’s t-test (n=3, *= pvalue<0.01; ** = pvalue < 0.01).

While senescent cells did not activate the ISR in response to stress, we did observe a senescence-specific increase in several transcripts, many of which in inflammatory pathways (Fig. 5A). The senescence associated secretory phenotype (SASP) is a senescence hallmark and describes the substantial secretion of cytokines, interleukins, and other proteins by senescent cells ^10,11,13^. Several SASP factors have also been associated with ER stress in different contexts ^50–52^ and we thus considered that while senescent cells do not effectively activate the ISR, stress may still increase SASP factor production.

To identify stress-induced increases comprehensively, we identified genes from our dRNA-seq data that were elevated after stress in Etop cells but not Cyc cells (Table S3). KEGG analysis confirmed that these genes were predominantly associated with inflammatory and secretory pathways (Fig. 5B). Though the SASP includes several proteins not typically associated with secretion^11^, we further refined our gene list to those annotated in the predicted secretome from the protein atlas ^53^. We found 21 secretory protein genes among the stress-enhanced genes, including several well-established SASP factors (Fig. 5C). Thus, our data indicate that while senescent cells do not effectively activate the ISR, they do transcriptionally enhance elements of the SASP during both proteotoxic and starvation stress.

In normal cells, activation of the ISR is followed by a recovery period after the initial stress has been resolved. This partly involves the action of the phosphatase GADD34 (PPP1R15A) that dephosphorylates eIF2α both to enable the translation of the upregulated factors and to begin the reduction of ATF4 expression^20^. In addition to PPP1R15A being a transcriptional target of ATF4, its translation is regulated using a similar inhibitory uORF mechanism^39^. Our model for deficient uORF bypass thus predicts that senescent cells would be unable to effectively express PPP1R15A. In fact, western blot analysis showed that PPP1R15A was inefficiently expressed during senescence even after treatment with Tg (Fig. 5D). This suggested that, in addition to being ineffective at increasing ATF4 protein levels, senescent cells may be unable to reduce the levels of eIF2α-P after stress. To test this, we treated Etop and Cyc cells with Tg for 3h, then changed the media and allowed cells to recover for an additional 20 hours in optimal growth conditions. Western blot analysis showed that the stressed Etop cells had marked increases in eIF2α-P compared to the untreated Etop cells, though neither condition showed enduring ATF4 expression (Fig. 5E). Interestingly, RT-qPCR analysis showed that the stress-enhanced transcriptome also lasted beyond the original stress, particularly for tested SASP factors (Fig. 5F). Thus, our results show that not only does stress enhance the senescent phenotype, but enhancement persists beyond the initial stress and optimal growth conditions are not sufficient to repair it.

## Discussion

**Figure 6.**
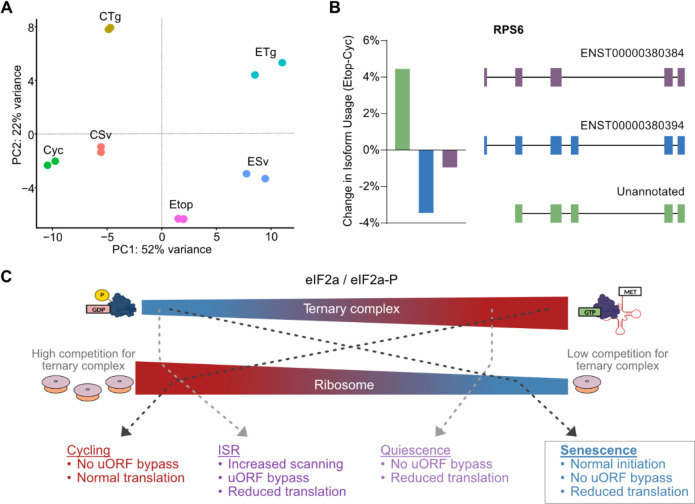
Model for translation repression and ISR inhibition in senescence. **(A)** Scatter plot of PC1 and PC2 from PCA of dRNA-Seq libraries. **(B)** Barplot of percent change in isoform usage and schematic of isoform structure for RPS6. **(C)** Model of reduced uORF bypass as a consequence of the imbalance of eIF2α/eIF2α-P ratio and ribosome abundance that results in persistent state of stress desensitization.

Senescence has been identified to serve multiple functions in organisms, from development to wound healing^32^, but is primarily expected to serve as a guard against tumorigenesis induced through sub-lethal genomic damage. As such, senescence can be viewed as a stress response, since it detects a stress and then induces an intense transcriptomic and proteomic remodeling for adaptation ^31,38,54^. The PCA for our dRNA-seq libraries point toward this same conclusion, wherein the first principal component appears to describe stress and places senescence as a more stressful state than exposure to either metabolic or proteotoxic stress (Fig. 6A). However, unlike the ISR, which functions to eventually restore homeostasis, senescence operates as a terminal stress response by converting damaged cells to an entirely new state with its own unique set of stable phenotypes. Thus, while principally like other stress responses, senescence has an entirely novel set of demands in its adaptive program, namely, to prepare for indefinite survival with unresolved cellular damage.

A defining feature of this framework is to achieve maximum survivability with minimal change, and thus avoidance of the ISR, even during stress. Our data show that the availability of translating ribosomes is a key determinant of senescent cells resistance to the ISR, and by extension to any stress response that requires proteomic remodeling. Even apoptosis, which senescent cells have long been understood to resist, would require substantial translational effort that our results show senescent cells appear to be intrinsically resistant to. A link between ribosome content and the efficacy of stress responses poses far-reaching implications to other conditions where ribosomes are dramatically decreased, such as ribosomopathies like Diamond-Blackfan anemia^55^.

The mechanism by which translating ribosomes are depleted in senescence may be due to a variety of sources. Senescent cells expand in size which may dilute the ability of ribosomes to adequately initiate^56^ and senescence has also been associated with decreases in rRNA transcription and maturation^41,42^. Interestingly, in addition to decreases in ribosomal proteins, our long-read sequencing data showed that ribosomal proteins frequently had increases in alternative splicing in senescence (Fig. 6B), though the contributions to protein levels are unclear. It is also possible that, rather than a reduction in absolute ribosome content, translation slows due to an accumulation of inactive 80S monosomes, which are stable but not bound to mRNA, and have been shown to arise in several conditions^57–59^. However, while the decrease in translating ribosomes is a defining difference between cycling and senescent cells, quiescent cells also reduce their translation capacity and have a similar aim to survive under sub-optimal conditions indefinitely. In fact, quiescence and senescence have several areas of commonality including the nature of their cell cycle arrest ^60^ and in the regulation of inflammatory genes by p38 MAPK-MK2 signaling ^61–63^. We further see that quiescent and senescent cells displayed high similarity in polysome profiling, further indicating a common translational arrest. What separates them in our work, is that neither contact-inhibition or serum-starvation induced quiescent cells show resistance to ISR activation as senescence.

The difference between quiescent and senescent cells may then be the degree of translational arrest that occurs. While quiescence is intended to be temporary and must therefore re-engage in the translationally expensive process of cell division, senescence is expected to be a terminal fate and so the translational capacity can be reduced to the minimum. Our results suggest that cell states can thus be modeled as a dynamic continuum of ribosome and ternary complex availability, wherein greater translation coincides with reduced ternary complex availability, which in turn increases stress sensitivity (Fig. 6C). One important question that arises from such a model is how these states are maintained and whether ribosome content is the cause or consequence of their induction. Future work will be needed to address how much of the senescence phenotype can be explained by decreased translation as well as understanding whether a restrictive translation environment poses liability to senescence that can be exploited therapeutically. Furthermore, since senescent cells are long-lasting and likely experience several rounds of stress, the way in which they cope with stress in the absence of stress responses will have important implications for aging.

## Methods

### Cell culture

IMR-90 human lung fibroblasts (Coriell Institute) were cultured in high-glucose DMEM (Gibco) supplemented with 10% heat-inactivated FBS (Gibco), nonessential amino acids (1x, Gibco), and antibiotic/antimycotic mix (1x, Gibco). Cells at ~40–60% confluency and at a population doubling level (PDL) between 20–30 were considered Cycling (Cyc). Contact-Inhibited Quiescent cells (Qui) were prepared essentially as described^64^ by allowing Cyc cells to reach ~100% confluency and growing for an additional four days, changing media every 2 days and 24 hours before harvest. Etoposide-induced senescent (Etop) cells were prepared by treating Cyc cells with 50 μM etoposide (Selleckchem, dissolved in DMSO) over 10 days (treatments on day 0, day 2, and day 4) with media changed every two days. Ionizing-radiation induced senescent (IR) cells were generated as described ^65^ by exposing Cyc cells to 15 grays of ionizing radiation from a cesium-137 irradiator (Nordion) and then culturing for 10 days with media changed every two days. Cells were harvested by trypsinization using TrypLE (ThermoFisher Scientific), pelleted at 4°C for 5 minutes at 600 × g and washed once in PBS before generation of protein lysates or bulk RNA as described below.

### Senescence validation

Senescence was validated using at least one of four methods: SA-β-Gal staining as per manufacturer’s instructions (Cell Signaling Technology), determination of cell cycle arrest using an EdU incorporation assay (ThermoFisher Scientific) as per manufacturer’s instructions, measurement of senescence associated protein markers (increased p16^INK4A^ and p21^CIP1^, decreased LMNB1), and elevated SASP factors measured by RT-qPCR (*CXCL8*, *IL6*, *CCL2*).

### Tandem Mass Tag mass spectrometry preparation and data analysis

Cyc, Qui, and Etop cells prepared as described were harvested by generating cell pellets through trypsinization and resuspended in mass spec buffer (100 mM Tris, 150 mM NaCL, 4% SDS, 1% Triton X-114, 0.1M DTT, and 1X Halt Protease Cocktail (Sigma) followed by sonication for 2.5 minutes and denaturation at 95°C for 3 minutes. Lysates were submitted for processing and analysis to Poochon Scientific (Frederick,MD) for quantitative proteomics using trypsin digestion, TMT-16plex labeling, fractionation by reverse-phase UHPLC, and LC-MS/MS analysis. Processed mass spectrometry data are available in Table S1.

Data was analyzed and volcano plots were generated using R data analysis software. Mass spectrometry data was filtered for proteins assigned with “medium” or “high” confidence at detection, as well as for proteins that showed a coefficient of variation ≤ 0.15 among biological triplicates. After filtering, principal component analysis was performed using base R function with all 9 sample libraries as the input. Biologically relevant protein changes were arbitrarily assigned to a log_2_ fold-change (log2FC) of 0.2/−0.2 and p-values ≤ 0.05 by two-tailed student’s T-test (Etop vs. Cyc, or Etop vs. Qui). GSEA analysis was performed on proteins identified as having at least 0.2/−0.2 log2FC in both the Etop/Cyc and Etop/Qui comparisons using the ClusterProfiler package^33^.

### Western blot and RT-qPCR analysis

Protein lysates for western blot analysis were extracted from cell pellets (≤ 2 × 10^6^ cells) using 100 uL lysis buffer (2% SDS 50 mM HEPES) followed by water bath sonication and denaturation at 95°C for 3 minutes. Lysates were quantified using a BCA protein assay kit (ThermoFisher Scientific) and then equal amounts of protein were loaded onto a 4–20% SDS-PAGE gel (Biorad) and then transferred to a nitrocellulose membrane (BioRad). Western blot analysis was performed using the iBind system (ThermoFisher Scientific) as described by the manufacturer and with antibodies and amounts listed in Table S4. Quantification of western blots was performed in Image Lab (BioRad). Antibodies and dilutions used in this study listed in Table S4.

Bulk-RNA free of genomic DNA for RT-qPCR analysis was extracted from cell pellets (≤ 2 × 10^6^ cells) using the RNeasy plus spin-column purification kit (Qiagen) and performed using the Qiacube liquid handler (Qiagen) as described by the manufacturer. Extracted RNA was then amplified using Maxima reverse transcriptase (ThermoFisher Scientific) and random hexamer primer. cDNA was then analyzed using two-step RT-qPCR using SYBR Green mix (Kappa biosystems) and analyzed using the 2^−ΔΔCT^ method. Primers used are listed in Table S4.

### Ribosome Sequencing Library Preparation

IMR90 cell prepared as described above for Cyc, Qui, IR, and Etop conditions were harvested by transfer to ice and washing once with ice-cold 0.1 mg/mL cycloheximide in PBS. After supernatant was removed completely, cells were lysed by adding 500 uL ice-cold polysome lysis buffer (20 mM Tris pH 7.5, 50 mM NaCl, 1 mM DTT, 50 mM KCl, 1% Triton x-100, 0.1 mg/mL cycloheximide, and 1x HALT protease phosphatase inhibitor cocktail (ThermoFisher Scientific)) dropwise. Lysates were scrapped and then triturated 3x through a 26g needle followed by centrifugation at 18k × g at 4°C for 10 min. RNA content was approximated by nanodrop OD 260 units and 50 ug was aliquoted for RNA-sequencing and Ribo-sequencing for each biological replicate.

Ribo-seq lysates were treated with 1 uL of RNAseq 1 (ThermoFisher Scientific) at 4°C for 40 minutes with shaking and reaction was stopped by addition of 200 U SUPERaseIN (ThermoFisher Scientific). Digested lysates were then overlaid on 900 uL of a 1M sucrose cushion in a 13 × 51 mm polycarbonate tube (Beckman Coulter), followed by ultracentrifugation at 70k RPM at 4°C for 2 h (Optima TLX ultracentrifuge, Beckman Coulter). Ribosome pellets were then resuspended in Trizol and extracted for RNA according to manufacturer’s instructions. Extracted RNA was then subjected to DNAse I treatment for 15 minutes at 37°C and then subjected to gel purification on a 15% polyacrylamide TBE-urea gel to isolate ribosome footprints. Isolated footprints were extracted from gel slices using extraction buffer (300 nM NaOAc pH 5.5, 1 mM EDTA, 0.25% SDS) overnight at room temperature followed by isopropanol precipitation. Precipitated footprints were depleted of rRNA using Low Input RiboMinus Eukaryote System v2 kit (ThermoFisher Scientific) and then subjected to end healing using T4 PNK (New England BioLabs). Resulting footprints were then used to generate sequencing libraries using the NEBNext Small RNA Library Prep Set for Illumina kit (New England BioLabs). Library quality was assessed using High Sensitivity DNA Chips with the Agilent 2100 Bioanalyzer System (Agilent Technologies).

RNA-seq lysates were extracted for bulk RNA in Trizol according to manufacturer’s instructions. RNA quality was assessed using a TapeStation analyzer (Agilent Technologies) and only RNA integrity numbers (RIN) greater than 9 were chose for further library preparation. Libraries were prepared with the TruSeq Stranded mRNA library preparation kit (Illumina).

Sequencing of Ribo-seq and RNA-seq libraries were performed in multiplex separately using S1 Flowcell for the NovaSeq 6000 sequencer (Illumina). Demultiplexing was performed using bcl2fastq software (Illumina).

### Ribosome Sequencing Analysis

FASTQ files for Ribo-seq reads were trimmed for adaptors (3’*AGATCGGAAGAGCACACGTCTGAACTCCAGTCAC*) using cutadapt (v4.0) and then filtered for rRNA reads by aligning against human18S, 28S, 5S, and 5.8S rRNA sequences using Bowtie2 v2.4.5 and collecting unaligned reads with the “–un” argument. FASTQ files for RNA-seq reads were also trimmed for adaptors (3’AGATCGGAAGAGCACACGTCTGAACTCCAGTCA) using cutadapt (v4.0).

Processed RNA-seq and Riob-seq reads were then aligned to the human genome (hg38 release, Ensembl GTF v108) using STAR (v2.7.10b) with “—quantMode” option enabled. Resulting count matrix was then used for translation efficiency analysis as previously described ^66^ using DEseq2 v1.36.0 and design argument as “~Condition+SeqType+Condition:SeqType”, where Condition was cell state (Cyc, Qui, Etop, IR) and SeqType was Ribo-seq or RNA-seq reads. RPKM analysis was obtained starting from the STAR generated read count matrix and then normalizing each gene tot total reads sequenced obtained from de-multiplexed fastq files prior to alignment; library-normalized reads were then transformed into RPKM by dividing reads by the max annotated transcript length (kb) for each gene.

### Treatment of cells with proteotoxic and metabolic stress

Starvation media (-Met -Cys) was prepared from high glucose DMEM lacking glutamine, methionine, and cysteine (Gibco) and then supplemented with 20% dialyzed FBS and glutamine to typical DMEM levels. Amino acid starvation was then performed by removing media from cultured cells, washing once in PBS, and then replacing with -Met -Cys media followed by incubation for 3h unless otherwise indicated. Proteotoxic stress was induced using thapsigargin (Tg, Sigma) supplemented into culture media described previously at 25 nM and incubated on cells for 3 h unless otherwise indicated. Cells for either stress condition were harvested after treatment and then prepared for bulk RNA or protein lysates as described above, or for direct-RNA nanopore sequencing as described below.

### Direct-RNA Nanopore sequencing

Total RNAs were extracted from cells grown in 150 mM plates using Trizol according to manufacturer’s instructions. RNA quality was measured using a Tapestation gel electrophoresis system (Agilent) and RNA integrity numbers greater than 8 were considered suitable for analysis. RNA was then prepared for sequencing essentially as described for TERA5-Seq ^46^. Briefly, ~40 μg of bulk RNA was subjected to poly(A) selection using Oligo d(T)25 magnetic beads (NEB) followed by on-bead 5’ ligation of a biotinylated REL5 linker sequence using T4 RNA ligase 1 (NEB) for 3 h at 37°C. 500 ng of REL5-ligated polyA RNAs were used for library preparation using the direct RNA sequencing kit (Nanopore Technologies). Libraries were quantified using a Quibit 1X dsDNA High Sensitivity assay kit (ThermoFisher Scientific) and then analyzed on a FLO-MIN106 flow cell on a Minion device (Nanopore Technologies).

### Processing and analysis of direct-RNA long-read sequencing

Direct RNA sequencing data were basecalled using Guppy (v3.4.5). By protocol design, some reads contain a 5’ linker (REL5) that marks the in-vivo 5’ end. Cutadapt (v2.8) was used to identify and remove adaptors ligated on the 5’ end of reads using parameters -g AATGATACGGCGACCACCGAGATCTACACTCTTTCCCTACACGACGCTCTTCCGATCT --overlap 31 --minimum-length 25 --error-rate 0. All reads except those smaller than 25nts are retained from further processing. Information about reads containing the adaptor is also retained for targeted analysis of 5’ end degradation. All reads were subsequently aligned against ribosomal sequences using minimap2 (v2.17). Reads that matched ribosomal sequences were excluded from further analysis. Filtered reads were mapped on reference human genome build 38 (hg38) using minimap2 (v2.17) with parameters -a -x splice -k 12 -u b -p 1 --secondary=yes. They were also aligned against the human transcriptome (Ensembl v91) with parameters -a -x map-ont -k 12 -u f -p 1 --secondary=yes. Transcript count was quantified as the number of reads aligning on each transcript. Differential expression of transcripts/genes was performed with DESeq2^67^.

### Poly-A tail and transcript length analysis

Nanopolish polya was used to extract poly(A) tail lengths from raw nanopore signal^68^. Only poly(A) tail lengths that passed the software quality control scores and were tagged as “PASS” were used in further analysis. Differential analysis for poly(A) tail and read lengths across conditions was performed with NanopLen package using the linear mixed model option for quantification of statistical significance (https://github.com/maragkakislab/nanoplen). In-house python scripts were used for data visualization.

### Transcriptome isoform analysis

Isoform analysis from direct RNA sequencing data was performed using FLAIR ^69^. All libraries were merged for isoform identification in order to create a common reference for all downstream comparisons. Differential isoform usage across conditions was then performed with diff_iso_usage.py and diffsplice_fishers_exact.py. Hits that were common in both replicate comparisons were selected as high confidence isoforms.

### Transfection of *in vitro* generated mRNA

cDNA for *in-vitro* generated mRNA constructs was obtained from expansion of an ATF4 Mammalian Gene Collection *Escherichia Coli* strain (Horizon Gene Editing, Clone ID: 3454473), while G. Extracted DNA was PCR amplified using primers listed in Table S4 to generate *ATF4* WT or Mut constructs, purified (PCR Cleanup Kit, Qiagen) and mRNA was generated using mMESSAGe mMACHINE T7 Ultra Kit (Ambion) according to manufacturer’s instructions. 500 ng of generated mRNA was transfected into Cyc or Etop cells using 4 μl Lipofectamine MessengerMAX Reagent (ThermoFisher Scientific). Cells were incubated for 12 hours until harvest for bulk RNA and protein extraction.

### Protein synthesis assay

Protein synthesis was measured using the Click-iT HPG Alexa Fluor 488 Protein Synthesis Assay kit (ThermoFisher Scientific). Cells were labeled in media as described above supplemented with 50 μM homo-paraglycine (HPG, methionine analogue) for times indicated in experiments. After labeling, cells were fixed and analyzed as per manufacturer’s instructions using a BZ-X (Keyence) fluorescent microscope. Protein synthesis was quantified by extracting the total brightness measured in the blue channel (fluorescein) from areas positive in the DAPI channel.

### Polysome profiling

Lysates for polysome profiling were prepared by treating cells with 100 μg/mL cycloheximide (Sigma) in culture media for 10 minutes at 37°C. Cells were then washed with ice-cold PBS also containing cycloheximide and lysed on ice by adding Polysome Buffer ( 20 mM Tris pH 7.4, 150 mM NaCl, 5 mM MgCl_2_, 1 mM DTT, 1 % v/v Triton X-100, 1x protease inhibitors (ThermoFisher Sceintific), 20 U/mL DNase I (Roche), 100 μg/mL cycloheximide) dropwise to cells and then scraping. Lysate was then triturated through a 26G needle (BD) and centrifuged for 10 minutes at 20k × g at 4°C. RNA content was approximated using UV absorbance on a Nanodrop machine and 20 μg of RNA was loaded onto 5%−45% sucrose gradient and analyzed on a UV/VIS fractionator (Brandell). Raw absorbance readings were analyzed and plotted in R and normalized UV absorbance was calculated by dividing each measurement over the total absorbance measured.

## Supplementary Material

Supplement 1

Supplement 2

Supplement 3

Supplement 4

Supplement 5

Supplement 6

## Data Availability

Sequencing data have been deposited in the Sequence Read Archive (SRA); accession: GSE223762 and GSE227766. Raw mass spectrometry data are available at the MassIVE data repository; accession: MSV000091077.
