# Supplementary material for: Senescence-specific translation dysregulation desensitizes cells to stress by inhibiting activation of the integrated stress response
Source: bioRxiv. 2023 Apr 12:2023.04.12.536613. Preprint. [Version 1] doi: 10.1101/2023.04.12.536613 (PMC10441410; doi:10.1101/2023.04.12.536613)
Supplement: Supplementary file 1 [file NIHPP2023.04.12.536613v1-supplement-6.pdf]

bioRxiv preprint doi: https://doi.org/10.1101/2023.04.12.536613; this version posted April 12, 2023. The copyright holder for this preprint (which was not certified by peer review) is the author/funder. This article is a US Government work. It is not subject to copyright under 17 USC 105 and is also made available for use under a CC0 license.

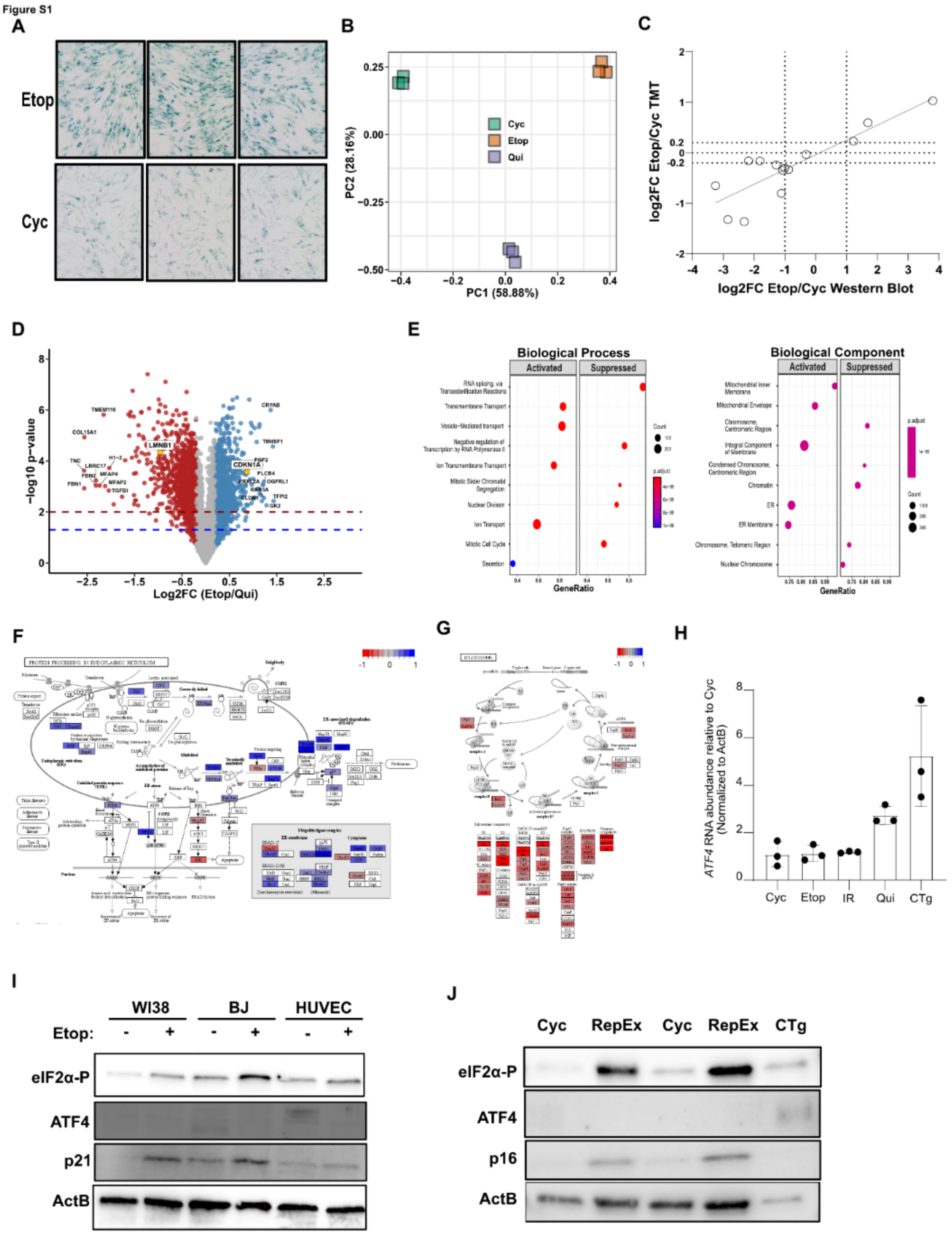

bioRxiv preprint doi: https://doi.org/10.1101/2023.04.12.536613; this version posted April 12, 2023. The copyright holder for this preprint (which was not certified by peer review) is the author/funder. This article is a US Government work. It is not subject to copyright under 17 USC 105 and is also made available for use under a CC0 license.

Figure S1. **(A)** Representative brightfield microscopy images of senescence-associated beta-galactosidase assay (n=6). **(B)** Principal component analysis of mass spectrometry data **(Table S1)**; boxes represent individual replicates for each cell state (n=3). **(C)** Correlation of log2FC measured by mass spectrometry and western blot analysis after normalization to ACTB for selected proteins (RPS20, p21, PCNA, LMNB1, eIF5, eIF2α, RPS10, eEF2, eEF2K, HSPA5, $p16^{INK4A}$, RPL14, eEF1A, eIF4EBP1). Dashed lines on y-axis correspond to cutoff used to determine substantial change in mass spectrometry data, dashed lines on x-axis correspond to log2FC of 1/-1. **(D)** Volcano plot of Etop/Qui differential protein expression analysis. Statistically significant (p-value ≤ 0.05 by two-tailed t-test) log2FC values are colored as follows: log2FC ≥ 0.2, blue; log2FC ≤ -0.2, red; log2FC between -0.2 and 0.2, grey. **(E)** Gene set enrichment analysis (GSEA) of proteins with uniquely increased and decreased expression in senescence. GSEA was performed for annotations in biological process (left) and cellular component (right) and p-value cutoff of 0.05. **(F-G)** KEGG pathway analysis of uniquely increased or decreased proteins visualized using Pathview[70]. Color indicates log2FC from mass spectrometry analysis. **(H)** RT-qPCR analysis for *ATF4* in indicated cell states (n=3). **(I)** Western blot analysis of Etop induced senescence in three additional cell types: WI-38 lung fibroblasts (WI38, 50μM etoposide), BJ human foreskin fibroblasts (BJ, 25μM etoposide), and Human Vein Endothelial Cells (HUVEC, 10μM etoposide). **(J)** Western blot analysis of replicative exhaustion induced senescent (RepEx) cells (PDL ~60).

bioRxiv preprint doi: https://doi.org/10.1101/2023.04.12.536613; this version posted April 12, 2023. The copyright holder for this preprint (which was not certified by peer review) is the author/funder. This article is a US Government work. It is not subject to copyright under 17 USC 105 and is also made available for use under a CC0 license.

Figure S2

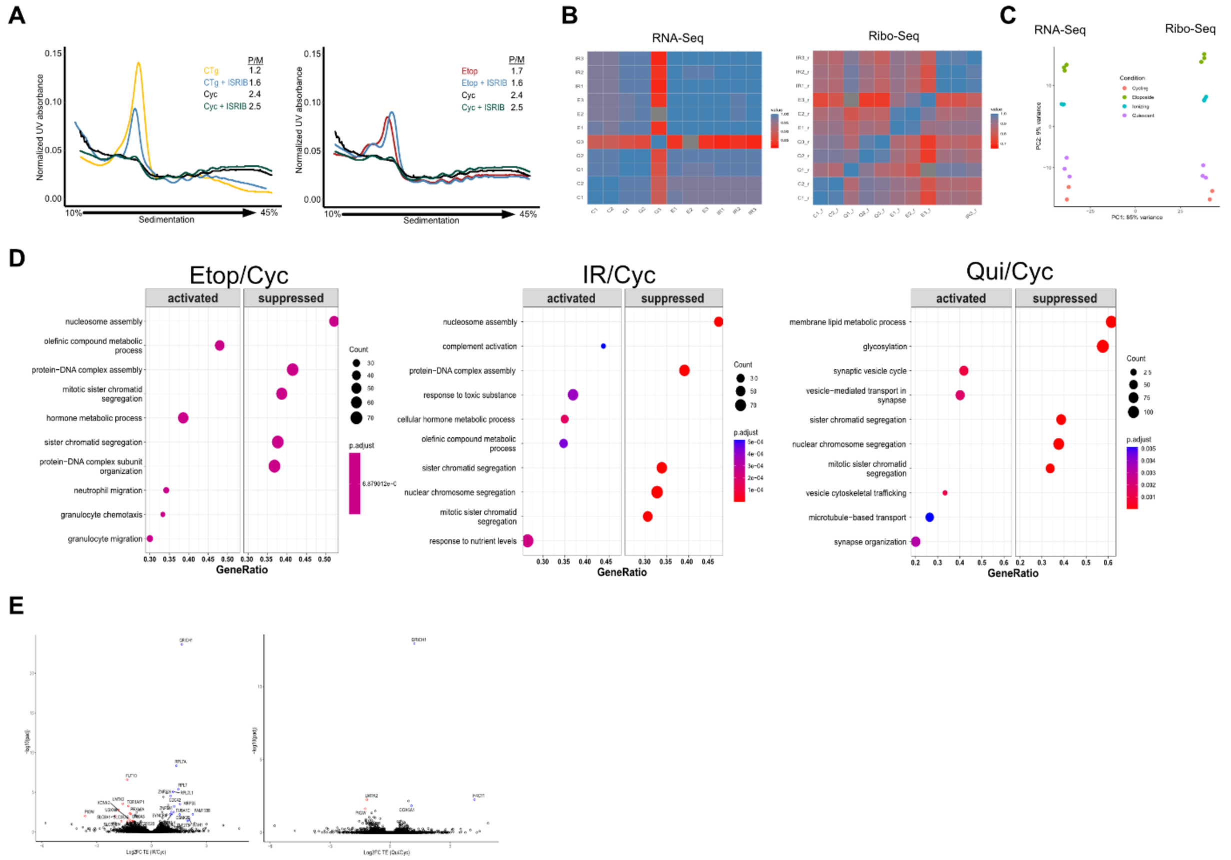

Figure S2. (A) Polysome profiling of cells treated with Tg (25 nM, 3h), ISRIB (10 nM, 24h) , or both. Translation rate assessed by polysome/monosome ratio (P/M). **(B)** Correlogram of RNA-seq and Ribo-seq libraries. **(C)** PCA of RNA-seq and Ribo-seq libraries; colors indicate cell type. **(D)** GSEA for biological process from RNA-seq data of indicated comparisons. **(E)** Volcano plot of differential translation efficiency for IR/Cyc (left) and Qui/Cyc (right) from ribosome sequencing. Statistically significant (adjusted p-value ≤ 0.05 by two-tailed t-test) log2FC values are colored as follows: log2FC ≤ -1, red; log2FC ≥ 1, blue; log2FC between -1 and 1, black.

bioRxiv preprint doi: https://doi.org/10.1101/2023.04.12.536613; this version posted April 12, 2023. The copyright holder for this preprint (which was not certified by peer review) is the author/funder. This article is a US Government work. It is not subject to copyright under 17 USC 105 and is also made available for use under a CC0 license.

Figure S3

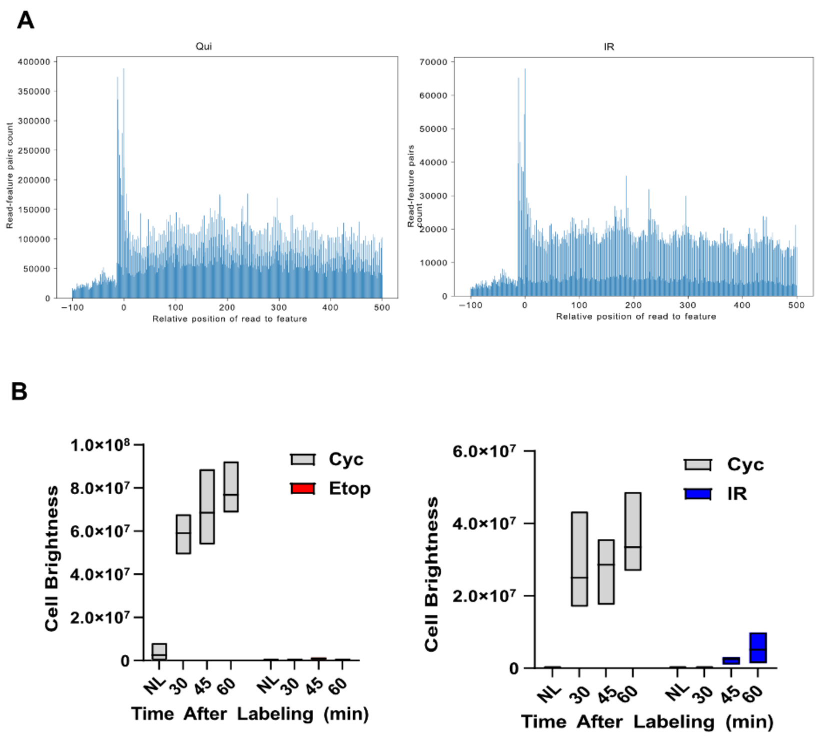

665

666 **Figure S3. (A)** Metaplot analysis of Ribo-seq reads mapped to the transcriptome. Reads are aligned relative to
667 annotated CDS start sites and are not normalized to gene length (positions are true nucleotide distances).

bioRxiv preprint doi: https://doi.org/10.1101/2023.04.12.536613; this version posted April 12, 2023. The copyright holder for this preprint (which was not certified by peer review) is the author/funder. This article is a US Government work. It is not subject to copyright under 17 USC 105 and is also made available for use under a CC0 license.

Figure S4

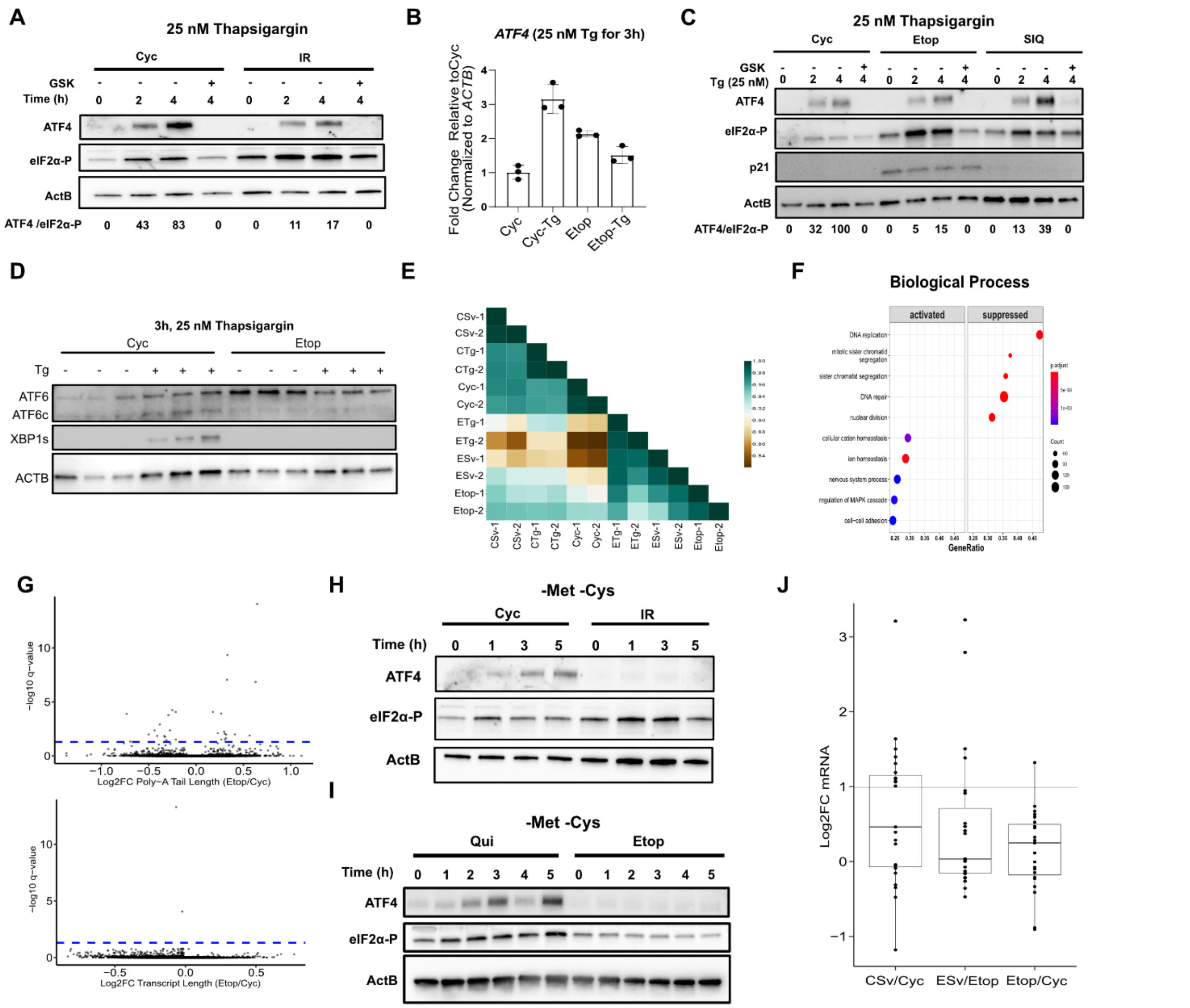

Figure S4. (A) Western blot analysis of Cyc and IR cells treated with 25 nM Tg at indicated times. ATF4/eIF2α-P ratio calculated using densiometric analysis of shown western blots and rounded to nearest integer; GSK (550 nM) added concurrently with Tg for indicated lanes. **(B)** RT-qPCR analysis for *ATF4* mRNA from bulk RNA extracted from Etop and Cyc cells with and without Tg treatment (3h, 25 nM). **(C)** Western blot analysis of Cyc, Etop and serum-induced quiescent (SIQ) cells treated with 25 nM for indicated times; GSK (550 nM) added concurrently with Tg for indicated lanes. SIQ cells were prepared by growing cells in media with 0.2% FBS for 10d. **(D)** Western blot analysis of Cyc and Etop cells treated with 250 ng/mL tunicamycin (Tm) for indicated times ; GSK (550 nM) added concurrently with (Tm) for indicated lanes. **(E)** Correlogram of dRNA-seq libraries. **(F)** GSEA for biological process of dRNA-seq Etop/Cyc comparison. **(G)** Volcano plots of log2 fold change for poly-A tail length (top) and transcript length (bottom) for Etop/Cyc dRNA-seq libraries obtained from NanopLen analysis (see methods). **(H-I)** Western blot analysis after starvation of methionine and cysteine amino acids cultured for indicated times. **(J)** Boxplot of dRNA-seq analysis for starvation genes[49]
